# Laparoscopic Cholecystectomy in Children: The Experience of Two Centers Focusing on Indications and Timing in the Era of “New Technologies”

**DOI:** 10.3390/children10111771

**Published:** 2023-10-31

**Authors:** Francesca Destro, Ugo Maria Pierucci, Eleonora Durante, Anna Maria Caruso, Vincenza Girgenti, Carlotta Paola Maria Canonica, Irene Degrassi, Alessandro Campari, Alessandro Pellegrinelli, Marta Barisella, Manuela Nebuloni, Marco Brunero, Elia Mario Biganzoli, Valeria Calcaterra, Gloria Pelizzo

**Affiliations:** 1 Department of Pediatric Surgery, Buzzi Children’s Hospital, 20154 Milan, Italy; francesca.destro@asst-fbf-sacco.it (F.D.); ugo.pierucci@asst-fbf-sacco.it (U.M.P.); eleonora.durante@asst-fbf-sacco.it (E.D.); carlotta.canonica@asst-fbf-sacco.it (C.P.M.C.); marco.brunero@asst-fbf-sacco.it (M.B.); 2 Pediatric Surgery Unit, Children’s Hospital, ARNAS Civico-Di Cristina-Benfratelli, 90127 Palermo, Italy; anna.caruso@arnascivico.it (A.M.C.); vincenza.girgenti@arnascivico.it (V.G.); 3 Department of Pediatrics, Buzzi Children’s Hospital, University of Milan, 20154 Milan, Italy; irene.degrassi@asst-fbf-sacco.it (I.D.); valeria.calcaterra@asst-fbf-sacco.it (V.C.); 4 Department of Pediatric Radiology, Buzzi Children’s Hospital, 20154 Milan, Italy; alessandro.campari@asst-fbf-sacco.it; 5 Pathology Unit, Department of Biomedical and Clinical Sciences, University of Milan, ASST Fatebenefratelli Sacco, 20157 Milan, Italy; alessandro.pellegrinelli@asst-fbf-sacco.it (A.P.); barisella.marta@asst-fbf-sacco.it (M.B.); manuela.nebuloni@unimi.it (M.N.); 6 Department of Biomedical and Clinical Sciences (DIBIC) & Data Science Research Center (DSRC), Unit of Clinical Research and Medical Statistics, Ospedale “L. Sacco” LITA Campus, University of Milan, 20122 Milan, Italy; elia.biganzoli@unimi.it; 7 Department of Internal Medicine, University of Pavia, 27100 Pavia, Italy; 8 Department of Biomedical and Clinical Science, University of Milano, 20157 Milan, Italy

**Keywords:** cholelithiasis, cholecystectomy, children, laparoscopy, surgical timing

## Abstract

Background: In children, laparoscopic cholecystectomy (LC) is now considered the gold standard for gallbladder (GB) removal. In the past, hemolytic disorders associated with cholelithiasis represented the most frequent conditions requiring LC; this is being overtaken by cholelithiasis and biliary conditions in overweight or ex-premature children. Aims: This study aims to describe current indications and timing for LC in pediatric patients. Methods: Retrospective study. Data on previous medical therapy, ultrasound, pre- and intraoperative aspects, and histology were collected for patients treated in 2020–2023. Results: In total, 45 patients were enrolled: 15 who underwent urgent surgery and 30 electives. Groups differed in terms of obesity rate, symptoms, ultrasound features, and intraoperative status. The most relevant risk factors for surgical complexity were age and pubertal stage, elevated cholestasis indexes, and gallbladder wall thickness > 3 mm at ultrasound. GB wall thickening ≥3 mm, US Murphy sign, fluid collections, and gallbladder distention on ultrasound correlated with high surgical scores. Conclusions: Indications for laparoscopic cholecystectomy in children seem to evolve caused by changing characteristics of the pediatric population. Patients with overweight/obesity may develop more complex GB diseases. Asymptomatic patients should be considered for surgery after observation, considering age and/or pubertal maturation when other risk factors are absent.

## 1. Introduction

Laparoscopic cholecystectomy (LC) in children is now considered the gold standard for gallbladder (GB) removal [1,2]. In the past, hemolytic disorders have represented the most frequent condition requiring LC. Pediatric cholelithiasis has been progressively increasing in the past decades and, to date, the trend is shifting towards cholelithiasis, biliary pancreatitis, cholecystitis, cholangitis, and, less commonly, biliary dyskinesia [1,3,4,5].

This trend is probably related to multiple factors, including the spread of childhood obesity and overweight and the survival of critical neonates and infants who received long-term medical care (parenteral nutrition and antibiotics) presenting sequelae of congenital malformation (e.g., duodenal atresia, biliary malformations) or severe conditions (e.g., short bowel syndrome) [6,7,8,9].

Despite the increased use of LC, many issues still need to be clarified, especially in children. The consensus needs to be greater regarding surgical timing for symptomatic and asymptomatic patients and more data should be available on the outcomes. Specific pediatric risk scores are required, since those used for adults have proven unreliable [1,7,10].

The best indications for surgical timing have been previously underlined [7]. The long duration of symptoms, systemic inflammatory signs, previous lithotherapy, and wall thickening ≥of 3 mm have been described as the major indications for immediate surgery. However, specific timing criteria were unavailable and clinical and auxological data needed to be included.

From a recent study, it is clear that the mean age has increased from 11 to 15.5 years and the mean BMI has increased from 19.2 cm/m^2^ to 23.0 cm/m^2^. Hereditary spherocytosis decreased from 63.6% to 11.8% of indications for cholecystectomy, while the proportion of cholesterol stones increased from 27.3% to 70.6% [8].

In this study, the scoring system proposed by Pelizzo et al. [7] was retrospectively applied for patients with cholecystic disease in order to evaluate its application in clinical practice and further explore the issue of surgical timing. We also highlight the benefits added by the application of new technologies, such as preoperative virtual reality (VR) three-dimensional (3D) models and indocyanine green (ICG) fluorescent cholangiography, to support the aim of the study.

## 2. Materials and Methods

### 2.1. Patients

From June 2020 to January 2023, patients admitted to two surgical departments of pediatric surgery (V. Buzzi Children’s Hospital, Milan, and ARNAS Civico-Di Cristina-Benfratelli, Palermo) with signs and symptoms of GB disease were prospectively enrolled in the study.

Clinical data, imaging details, surgical procedures, histological results, and outcomes were recorded. An adaptation of the scoring system previously described [7] was applied, and each patient’s severity was determined based on the overall score obtained. The detailed scoring system can be found in the Supplementary Material.

Only data of pediatric patients (age < 18) undergoing LC were considered for the analysis. Urgent laparoscopic surgery (ULS) was performed in patients with complicated cholelithiasis, no symptom resolution, and no biochemical changes in inflammation (according to the 2018 Tokyo Guidelines) within seven days of symptom onset [11]. This group included patients with right upper quadrant mass/pain/tenderness, Murphy’s sign, and systemic signs such as fever, elevated C-reactive protein, and white blood cell count. Those patients who did not fall within these urgency criteria were electively scheduled (ELS, elective laparoscopic surgery) after 3–6 months of conservative treatment with ursodeoxycholic acid (dose 20 mg/kg/day), clinical examination, and US evaluation approximately once a month. Associated hematological disease and multiple stones detected on the US were considered as indications for surgery for asymptomatic patients.

Data have been retrospectively evaluated according to the principles of the Declaration of Helsinki as revised in 2008. Ethical committee approval was not requested because the General Authorization to Process Personal Data for Scientific Research Purposes (Authorization no. 9/2014) declares that ethical approval is not needed for retrospective archive studies that use ID codes, preventing the data from being traced back directly to the data subject. The reservedness of the collected information was ensured according to Regulation (EU)/2016/679 GDPR (Regulation (EU) 2016/679), Legislative Decree n.101/18.

The primary outcome of this study was to evaluate the application of the Pelizzo scores [7] in patients with cholecystic disease and surgical indications. In order to support this primary aim, the secondary outcome included the critical analysis of surgical timing and the benefits of applying new technologies.

#### 2.1.1. Clinical Data

Data on epidemiology (age, gender, ethnicity), medical and family history, perinatal data (gestational age and birth weight), associated medical conditions, previous medical/surgical therapy, and anamnesis on the symptoms’ onset, type, and duration were collected. Weight was evaluated standing upright in the center of the scale platform (Seca, Hamburg, Germany) [12]. Height was measured using a Harpenden stadiometer with a fixed vertical backboard [12]. BMI was calculated as body weight (kilograms) divided by height (meters squared). According to WHO classification, children aged between 5–19 years are classified as overweight or with obesity when body mass index (BMI) for age and sex is at or above the 85th percentile and below the 97th percentile, or above the 97th percentile, respectively [13]. Pubertal stages were collected at time of surgery, classified according to Marshall and Tanner [14], and considered as follows: prepubertal/early puberty (PRE/EA-Puberty) = Tanner stage 1–2; middle/late puberty (MI/LA-Puberty) = Tanner stages 3–5.

#### 2.1.2. Radiological Data

All patients underwent sonographic examination for detection of specific items:

– presence, maximum diameter, location, and mobility of gallstones;
– appearance, volume, and diameters of the gallbladder (GB, e.g., wall thickening, pericholecystic fluid);
– features of the biliary tree (e.g., dilatations and presence of calculi);
– presence of hepatic/splenomegaly or hepatic steatosis;
– presence of lymph nodes in the hepatic pedicle and sonographic Murphy sign (maximal tenderness from US probe pressure over the GB).

In complicated cases, MRI with cholangiographic sequences was performed before surgery to precisely assess biliary and vascular anatomy. The radiological images were elaborated to obtain 3D models that were zoomable and viewable from many viewpoints and hidden or shown in transparency, allowing focus on specific structures. Free, open-source software was used for image segmentation (https://www.slicer.org, accessed on 23 August 2022) and 3D models were loaded into an HMD (head-mounted display) (Oculus Quest v.1—META Inc., Menlo Park, CA, USA) [15,16].

#### 2.1.3. Surgical LC

LC was performed with the standard four-trocar technique as previously described [7], by operators with more than five years of experience in the two centers. Surgical details were described: the presence of adhesions (> or <50%), the aspect of the gallbladder (distended/contracted, unable to be grasped), the impact of stones, the presence of inflammation signs, and the time to identify the cystic artery/duct (> or <90 min).

In cases of suspected or certain choledocholithiasis, endoscopic retrograde cholangiopancreatography (ERCP) or intraoperative cholangiography with laparoscopic common bile duct exploration (LCBDE) were planned before or during the operation, respectively.

ICG fluorescent cholangiography using RUBINA^TM^ technology (KARL STORZ SE & Co KG, Tuttlingen, Germany) has been recently adopted as surgical guidance to define the extrahepatic biliary anatomy. Patients receive an intravenous ICG injection (0.35 mg/kg) the day before surgery and during surgery, and the ICG near-infrared fluorescence (NIRF) image allows a real-time fluorescent visualization of the extrahepatic biliary tree to guide the surgical dissection.

#### 2.1.4. Histological Examination

Pathologists with pediatric experience examined all the removed gallbladders. The specimens were fixed in formalin and embedded in paraffin, and 3-micron sections were cut and stained with hematoxylin and eosin. The histological parameters analyzed were ulcers/erosions, inflammatory cell infiltration, fibrosis, adenomyosis, reactive epithelial hyperplasia, epithelial atrophy, parietal atrophy, intramural micro-lithiasis, and intestinal metaplasia. As for US and surgery, a histopathological severity score was obtained (one or zero points were assigned for the presence or absence of the abovementioned histological features, respectively).

### 2.2. Statistical Analysis

The normality distribution of the variables was tested with the Shapiro–Wilk test. Categorical variables were described as frequencies and percentages, and continuous variables were expressed as the mean (±standard deviation, SD) or the median and IQR (interquartile range) as appropriate. We used the Fisher test to analyze categorical variables and the Student’s *t*-test for continuous variables or the Wilcoxon rank-sum test as appropriate. Surgical time and surgical risk were considered as dependent variables for two respective univariate linear regression models using the following as predictive variables: age, sex, prematurity, family history of cholelithiasis, obesity, symptoms, cholestasis, hematological diseases, gallbladder wall >3 mm, distended gallbladder on US, US Murphy sign, stone diameter, gallbladder fluid collections on US.

The dependent variables for the three respective logistic regression models were the presence of adhesions >50%, intraoperative inflammation signs, and gallbladder appearance (contraction vs. distension). Univariate variable selection (likelihood-ratio test) using *p* < 0.25 to select candidates for the multivariable model was performed on the same variables as the linear regression. In building the final model, *p* < 0.05 was considered statistically significant.

Data were analyzed with Stata 18.0 BE (Stata Corporation, College Station, TX, USA).

## 3. Results

Forty-five patients (23 F/22 M) met the inclusion criteria and were enrolled in the study. Family history of cholelithiasis was reported in 12 patients (26.6%). Sixteen patients (35.5%) reported an associated medical condition, including hematological diseases such as spherocytosis (seven cases, 15.5%), genetic alterations (6.6%: one case of PRSS1 gene mutation, one Gilbert syndrome, and one trisomy 21), hepatic steatosis (three patients, 6.6%), duodenal atresia with altered anatomy (one patient, 2.2%) and others (ligament laxity, hypothyroidism). In total, 41 patients were symptomatic (41/45, 91.1%).

The mean age at surgery was 12.3 ± 3.3 years (age range 2–16 years). Overall, 62% of cases (29 patients) were MI/LA-Puberty; three were born premature and seven had a BMI >85th percentile. Most MI/LA-Puberty patients had multiple stones detected on the US (23/28 cases) and US signs such as GB thickness >3 mm (11/28 cases) and peri-cholecystic collections (9/28 cases). During surgery, 17/28 patients had adhesions >50% and 5/28 had stone impact; 19/28 showed signs of infection and in 5/28 the operative time to detect the cystic artery/duct was >90 min.

Sixteen patients (35.5%) received surgery at PRE/EA puberty stage. No differences between the two puberty groups were evident in terms of symptoms (*p* = 0.11), complicated cases with need for ERCP (*p* = 0.39), and post-operative complications (*p* = 1.00), as shown in Table 1.

Seven patients (15.5%) had a BMI for age and sex above the 85th percentile and most of them showed MI/LA-Puberty stage (*p* = 0.032). All overweight patients received ULS with a significant difference compared with the ELS group (*p* < 0.001). Nevertheless, overweight/obesity was not related to the need for ERCP (*p* = 0.30) nor the development of postoperative complications (*p* = 0.41), as shown in Table 1.

Fifteen patients (33.3%) received ULS, whereas the remaining 30 patients (66.6%) were scheduled for ELS. Cholelithiasis was the commonest indication for LC (24 cases, 53.3%), followed by cholecystitis (7 cases, 15.5%), hematological disorders (7 cases, 15.5%), pancreatitis (4 cases, 8.8%), cholecystitis and pancreatitis together (1 case, 2.2%), gallbladder duplication (1 case, 2.2%), and gallbladder polyps (1 case, 2.2%). Cholestasis was identified in 15 cases (33.3%), 8 requiring ULS and 7 ELS (*p* = 0.044). No significant differences between gender (*p* = 0.14) and family history of cholelithiasis (*p* = 0.47) were identified between the groups. Ethnicity and onset of puberty were also irrelevant.

Biochemical parameters were similar in both groups, without differences for each considered variable (*p* > 0.05).

Considering sonographic features, the US Murphy sign (*p* = 0.009) and gallbladder wall >3 mm (*p* = 0.016) were more common in the ULS group (Table 2). At the same time, there were no differences regarding number of stones (*p* = 0.90), stone diameter (0.13), nor gallbladder distension (*p* = 0.53); the latter is a more frequent sign in children with overweight/obesity compared to normal-weight patients (*p* = 0.016, Table 2). The univariate analysis showed a high significance between fluid collections on the US and the surgical risk score (*p* < 0.001).

A 3D reconstruction of preoperative MRI was performed in two patients. One of them underwent surgery in the neonatal period for duodenal atresia, and years later she developed symptomatic pancreato-biliary tree stones. The 3D models clearly showed the stones’ disposition and peculiar anatomy (the choledochal channel ends in the upper duodenal stump with dilatation and the pancreatic channel goes to the lower duodenal stump). The other case was a boy with stones and genetic-based chronic pancreatitis who required multiple endoscopic procedures before LC to obtain biliopancreatic drainage and symptom relief after acute and severe abdominal pain attacks.

ERCP was attempted before surgery in seven cases (15.5%); it was successful in six cases and was technically unfeasible due to difficulty reaching the papilla in one patient who immediately underwent LC and anterograde cholangiography with papilla dilatation. Intraoperative cholangiography with or without LCBDE was performed as a primary procedure on 13 patients (28.8%). Adhesions > 50% were more common in the ULS group (*p* = 0.05). Other surgical macroscopic features did not differ between ULS and ELS (gallbladder distension/contraction *p* = 0.46, stone impact *p* = 0.41, signs of inflammation *p*= 0.33, time to identify the cystic artery/duct > 90 min *p* = 0.41). Body weight did not influence surgery.

**Figure 1:**
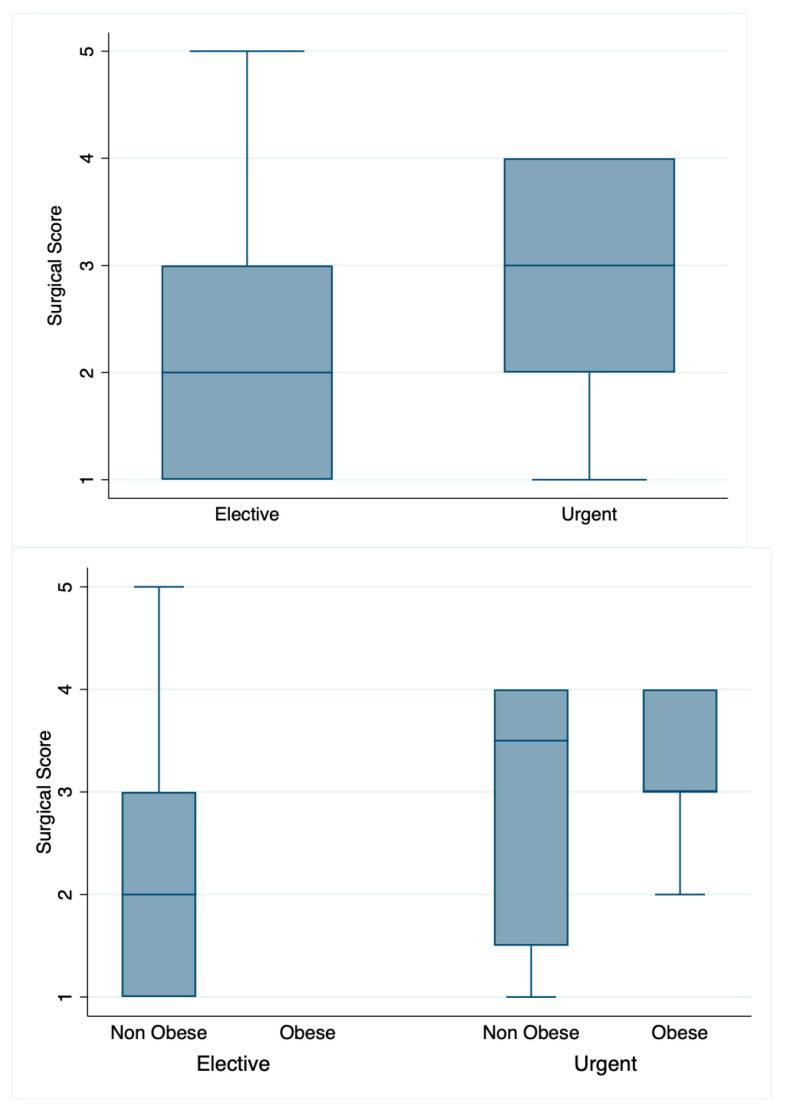
Graphic distribution of variables.

No significant correlation was found between surgical score and timing (urgent vs. elective) nor between the surgical score and BMI (normal weight vs. overweight/obesity, Table 3 and Figure 1).

Univariate logistic analysis for the presence of adhesions >50% identified the presence of cholestasis as a predictive factor (OR 4.12; 95% CI: 1.06–16.03; *p* = 0.04); multivariate logistic analysis for gallbladder contraction identified blood diseases and stone diameter as predictors (blood diseases: OR 9.75; 95% CI: 1.22–77.7; *p* = 0.032; stone diameter: OR 0.07; 95% CI: 0.01–0.51; *p* = 0.008); multivariate logistic analysis for gallbladder distention identified age and distended gallbladder on US as predictors (age: OR 1.4; 95% CI: 1.06–2.07; *p* = 0.018; distended gallbladder on US: OR 8.46, 95% CI: 1.18–60.36; *p* = 0.033); univariate logistic analysis for intraoperative inflammation signs identified gallbladder wall >3 mm on US as a predictive factor (OR 13.59; 95% CI: 1.57–117; *p* = 0.018).

Five patients underwent ICG fluorescent cholangiography during surgery (11%; one ULC and four ELC).

Any conversions or intraoperative complications were reported. Three patients developed complications after surgery (two grade 1 and one grade 3, according to Clavien–Dindo, Table 1) with no differences between ULS and ELS (*p* = 0.20).

Histological parameters did not differ in patients who received emergency versus elective surgery. On the other hand, adenomyosis (*p* = 0.046) and reactive epithelial hyperplasia (*p* = 0.005) were more frequent in patients with overweight/obesity (Table 4).

## 4. Discussion

We applied the Pelizzo scores [7] to patients with cholecystic disease managed in two pediatric surgical centers in order to describe current indications and timing for LC in pediatric patients. Our results show that the main surgical indication was symptomatic cholelithiasis and a discrete percentage of patients required an urgent approach.

Patients with overweight/obesity showed a prevalence of MI/LA-Puberty stage and mostly received ULS without differences in complication rates. On the other hand, their histological examinations revealed higher rates of adenomyosis and reactive epithelial hyperplasia. Considering the pubertal stages, MI/LA seems associated with sonographic and surgical severity elements with no implications regarding symptoms and complications.

GB wall > 3 mm on US, US Murphy sign, US fluid collections, cholestasis, older age at surgery, and blood diseases can be considered as surgical risk factors.

LC is a well-established approach for gallbladder removal in the adult population, and its application in children has been increasing in recent decades [9,17] with cholelithiasis as its most common indication [18,19,20].

Clinical manifestations of cholelithiasis are highly variable, from completely asymptomatic children (80%) to patients with nonspecific mild symptoms or severe clinical cholecystitis, cholangitis, and pancreatitis [7,21]. This variability can make it difficult to indicate surgery and define the timing [22].

ELS is recommended in patients with hemolytic anemia without specific haste [23]. It is usually performed simultaneously with the splenectomy, but it gives advantages even afterward [23]. The association of cholecystectomy and splenectomy probably explains the younger age of the spherocytosis patients in our series. Compared with older patients, the similar rate of complications confirms the procedure’s safety.

Surgery should also be scheduled for symptomatic patients who do not respond to medical therapy or in case of complication, whereas a period of conservative management can be proposed for asymptomatic patients. The rationale for waiting is based on the possibility that these patients remain asymptomatic even as they grow or that the stones disappear [22,24]. However, they could develop chronic and complicated diseases without an apparent clinical picture. A recent study on 22.257 adult patients with asymptomatic gallstones showed that the development of symptoms occurs at approximately 2% per year, especially in the presence of the following risk factors: female gender, younger age, multiple stones, GB polyps, large stones, and chronic hemolytic anemia [25]. Children seem to be subject to the effects of recurrent mild or subclinical episodes of inflammation leading to severe gallbladder damage and pre-cancerous conditions [7]. Still, the definition of timing can be complex [7,9,22].

We should consider that the severity of the pathology is the main influencer of the therapeutic choices, affecting the results [26,27,28]. In adults, radiological and clinical parameters define the severity and, thus, the surgical timing [29,30], but they do not apply to children [7]. A recent paper by our coauthors showed that long duration of symptoms, systemic inflammatory signs, previous lithotherapy, and wall thickening ≥3 mm should be considered indicators of severe forms. At the same time, age, sex, and history of abdominal surgery are not useful [7]. Our results are consistent with these data. Considering the sonographic signs, we found that GB wall thickening ≥3 mm, US Murphy sign, and fluid collections are associated with more severe forms. In contrast, the characteristics of the stones (diameter and number) seem to be irrelevant. In a previous study, small stones (at least one < 5 mm in diameter) increased the risk of developing complicated GB disease, but this association was not confirmed by Kirsaclioglu et al., who identified older age, independent of stone size and etiology, as a risk factor [23,31]. We also found that older age at surgery, cholestasis, hematological diseases, GB wall thickening >3 mm, and GB distension in the US are risk factors for a more complex surgical procedure.

Obesity is a common risk factor for cholelithiasis, with rising rates in children [23]. The prevalence of cholelithiasis in children and adolescents with obesity grows from 0.13–0.3% to 2–6.1% [23]. Greer et al. addressed the phenomenon as a “facet of the obesity epidemic” [32]. In our series, patients with overweight/obesity more often required ULS and presented multiple sonographic risk signs. We should take this as a warning to consider overweight/obesity as a complex, multifaceted disease requiring the involvement of many pediatric health practitioners.

As previously demonstrated, LC appeared to be a safe approach for emergency and elective surgery [23]. The presence of multiple adhesions in ULC, as reported, could complicate the procedure and represent a risk factor for developing complications. Our work could not demonstrate correlations between surgery regimen (elective versus urgent) and surgical times or complication rates, as could be expected, possibly because of a relatively small cohort size.

In case of complicated GB disease (choledocholithiasis, common bile duct dilatation, gallstone pancreatitis), ERCP is indicated to drain the biliary tree or to remove stones after sphincterotomy [33]. Recently, the literature suggests performing LCBDE instead of ERCP to provide a definitive treatment in a single procedure and reduce complications associated with the endoscopic operative approach (ERCP complication risk is 5–10%) [34,35,36]. Although there are conflicting previous reports, the pediatric DUCT criteria (common bile duct dilation, US choledocholithiasis, and total bilirubin ≥1.8 mg/dL) seem to estimate the risk of choledochal involvement with high accuracy (>76%), specificity (>78%), and negative predicted values (>79%) [10].

Histological examination of the specimen is essential in caring for children with GB pathologies to detect metaplasia [7]. Although elective versus emergency procedures did not show differences in histological parameters, we identified more frequent elements in overweight/obese patients (adenomyosis and reactive epithelial hyperplasia), confirming that BMI is a risk factor for developing microscopic changes.

Although our experience is limited, recent technological and educational innovations promise to extend treatment options for children with complex GB pathologies. Three-dimension models used for preoperative surgical simulations help understand the anatomy’s complexity [15]. ICG fluorescent cholangiography is a real-time surgical aid to target the anatomy, quickly obtaining the “critical view of safety” [28,37]. A better comprehension of biliary, duodenal, and pancreatic anatomy needs pre- and intraoperative imaging support to reduce the intraoperative risk and ameliorate the patient’s outcome. Children with congenital malformation or a suspicion of pancreatic disease and/or malformation should benefit from these technologies.

Some limitations should be acknowledged. First, this study’s retrospective nature may determine biases in the statistical analysis and reduce the amount and type of information available for each patient. Perspective and multicenter studies would improve the results’ quality and support this study’s validity. Second, the surgical timing was not randomized, and management is based on medical clinical decisions made by pediatric surgeons, reflecting the decision-making process but introducing a potential limitation. The effects of overweight/obesity and the correlation with stones should be investigated on a broader range of premature infants to better determine each variable’s role. The joint effort must continue toward the validation of clinical and ultrasound scores and the development of new technologies. Identifying clinical and/or radiological risk factors may help better define surgical timing and indications for asymptomatic patients.

## 5. Conclusions

Surgical indications for pediatric patients with cholelithiasis include complicated cases and hematologic diseases. Asymptomatic patients may be considered for surgery after an adequate observation period, but the need for an operation in these children is yet to be confirmed.

Overweight and obesity may have negative repercussions on surgical procedures and histological results. These data underline the importance of their early management by a dedicated multidisciplinary team and the possible need for early surgery.

Preoperative sonographic evaluation supports the surgical choices, easily identifying factors associated with a high surgical score, such as GB wall thickening ≥3 mm, US Murphy sign, and fluid collections.

## Figures and Tables

**Table 1:** Clinical features in our series and the four subgroups (PRE/EA-Puberty and MI/LA-Puberty; normal weight and overweight/obese).

Clinical Features								
		Total	PRE/EA-Puberty	MI/LA Puberty	*p*-Value	Normal Weight	Overweight/Obese	*p*-Value
N		45	16	29		38	7	
Age median (IQR)		13.0 (11.0–14.0)	9.0 (7.5–11.0)	14.0 (13.0–16)	<0.001	12.5 (10.0–14.0)	13.0 (12.0–14.0)	0.27
Male		22 (49%)	10 (62%)	12 (41%)	0.22	20 (53%)	2 (29%)	0.41
Family history of cholelithiasis		12 (27%)	4 (25%)	8 (28%)	1.00	10 (26%)	2 (29%)	1.00
Obesity		7 (16%)	0 (0%)	7 (24%)	0.04			
Symptoms					0.11			0.24
	Asymptomatic	4 (9%)	1 (6%)	3 (10%)		4 (11%)	0 (0%)	
	Pain	30 (67%)	14 (88%)	16 (55%)		26 (68%)	4 (57%)	
	Pancreatitis	3 (7%)	1 (6%)	2 (7%)		2 (5%)	1 (14%)	
	Cholecystitis	7 (16%)	0 (0%)	7 (24%)		6 (16%)	1 (14%)	
	Cholecystitis and pancreatitis	1 (2%)	0 (0%)	1 (3%)				
Surgery					0.19			<0.001
	Urgent	15 (33%)	3 (19%)	12 (41%)		8 (21%)	7 (100%)	
	Elective	30 (67%)	13 (81%)	17 (59%)		30 (79%)	0 (0)	
Spherocytosis		7 (16%)	5 (31%)	2 (7%)	0.079	7 (16%)	0 (0%)	1.00
Cholestasis		15 (33%)	6 (38%)	9 (31%)	0.75	11 (29%)	4 (57%)	0.20
ERCP		7 (16%)	1 (6%)	6 (21%)	0.39	5 (13%)	2 (29%)	0.30
Post-operative complications		3 (7%)	1 (6%)	2 (7%)	1.00	2 (5%)	1 (14%)	0.41

PRE/EA-Puberty = prepubertal/early puberty; MI/LA-Puberty = middle/late puberty; ERCP = endoscopic retrograde cholangiopancreatography.

**Table 2:** Sonographic results.

Sonographic Signs								
		Total	ELS	ULS	*p*-Value	Normal Weight	Overweight/Obese	*p*-Value
N		45	30	15		38	7	
Number of stones					0.90			1.00
	0	1 (2%)	1 (3%)	0 (0%)		1 (3%)	0 (0%)	
	1	7 (16%)	4 (13%)	3 (20%)		6 (16%)	1 (14%)	
	2	3 (7%)	2 (7%)	1 (7%)		3 (8%)	0 (0%)	
	Multiples (≥3)	34 (76%)	23 (77%)	11 (73%)		28 (74%)	6 (86%)	
US Murphy sign		26 (58%)	13 (43%)	13 (87%)	0.009	19 (50%)	7 (100%)	0.016
GB wall > 3 mm		13 (29%)	5 (17%)	8 (53%)	0.016	7 (18%)	6 (86%)	<0.001
Stone diameter > 3 mm		35 (78%)	21 (70%)	14 (93%)	0.13	28 (74%)	7 (100%)	0.32
GB distention		23 (51%)	14 (47%)	9 (60%)	0.53	17 (45%)	6 (86%)	0.096
GB fluid collections		12 (27%)	5 (17%)	7 (47%)	0.070	8 (21%)	4 (57%)	0.069

ELS = elective laparoscopic surgery; ULS = urgent laparoscopic surgery; US = ultrasound; GB = gallbladder.

**Table 3:** Intraoperative macroscopic features.

Intraoperative Macroscopic Features								
		Total	ELS	ULS	*p*-Value	Normal Weight	Overweight/Obese	*p*-Value
N		45	30	15		38	7	
Adhesions > 50%		23 (51%)	12 (40%)	11 (73%)	0.05	18 (47%)	5 (71%)	0.41
GB distention		35 (78%)	22 (73%)	13 (87%)	0.46	29 (76%)	6 (86%)	1.00
GB contraction		10 (22%)	8 (27%)	2 (13%)	0.46	9 (24%)	1 (14%)	1.00
Stone impact		8 (18%)	4 (13%)	4 (27%)	0.41	6 (16%)	2 (29%)	0.59
Signs of inflammation		27 (60%)	16 (53%)	11 (73%)	0.33	21 (55%)	6 (86%)	0.22
Time to identify cystic artery/duct > 90 min		8 (18%)	4 (13%)	4 (27%)	0.41	6 (16%)	2 (29%)	0.59
Surgical score					0.072			0.12
	0	0	0	0		0	0	
	1	15 (33%)	13 (43%)	2 (13%)		15 (39%)	0 (0%)	
	2	7 (16%)	5 (17%)	2 (13%)		6 (16%)	1 (14%)	
	3	12 (27%)	7 (23%)	5 (33%)		8 (21%)	4 (57%)	
	4	9 (20%)	3 (10%)	6 (40%)		7 (18%)	2 (29%)	
	5	2 (4%)	2 (7%)	0 (0%)		2 (5%)	0 (0%)	
	6	0	0	0		0	0	

ELS = elective laparoscopic surgery; ULS = urgent laparoscopic surgery; GB = gallbladder.

**Table 4:** Histological results for the removed specimens.

Histological Parameters											
		Total	ELC	ULC	*p*-Value	Normal Weight	Overweight/Obese	*p* Value	PRE/EA-Puberty	MI/LA-Puberty	*p*-Value
	N	45	30	15		38	7		16	29	
Ulcers and/or erosion		13 (29%)	6 (20%)	7 (47%)	0.086	9 (24%)	4 (57%)	0.17	1 (6%)	12 (41%)	0.016
Inflammatory cell infiltration		40 (89%)	27 (90%)	13 (87%)	1.00	34 (89%)	6 (86%)	1.00	15 (94%)	25 (86%)	0.64
Fibrosis		23 (51%)	15 (50%)	8 (53%)	1.00	18 (47%)	5 (71%)	0.41	6 (38%)	17 (59%)	0.22
Adenomyosis		4 (9%)	1 (3%)	3 (20%)	0.10	2 (5%)	2 (29%)	0.11	1 (6%)	3 (10%)	1.00
Reactive epithelial hyperplasia		23 (51%)	13 (43%)	10 (67%)	0.21	16 (42%)	7 (100%)	0.009	7 (44%)	16 (55%)	0.54
Epithelial atrophy		16 (36%)	12 (40%)	4 (27%)	0.51	14 (37%)	2 (29%)	1.00	3 (19%)	13 (45%)	0.11
Parietal atrophy		11 (24%)	8 (27%)	3 (20%)	0.73	9 (24%)	2 (29%)	1.00	2 (12%)	9 (31%)	0.28
Intramural microlitiasis		20 (44%)	15 (50%)	5 (33%)	0.35	17 (45%)	3 (43%)	1.00	6 (38%)	14 (48%)	0.54
Intestinal metaplasia		0 (0%)	0 (0%)	0 (0%)		0 (0%)	0 (0%)		0 (0%)	0 (0%)	
Histological Score					0.76			0.79			0.22
	0	3 (7%)	3 (10%)	0 (0%)		3 (8%)	0 (0%)		1 (6%)	2 (7%)	
	1	7 (16%)	4 (13%)	3 (20%)		7 (18%)	0 (0%)		5 (31%)	2 (7%)	
	2	7 (16%)	4 (13%)	3 (20%)		6 (16%)	1 (14%)		3 (19%)	4 (14%)	
	3	6 (13%)	5 (17%)	1 (7%)		5 (13%)	1 (14%)		3 (19%)	3 (10%)	
	4	6 (13%)	4 (13%)	2 (13%)		5 (13%)	1 (14%)		1 (6%)	5 (17%)	
	5	10 (22%)	7 (23%)	3 (20%)		8 (21%)	2 (29%)		1 (6%)	9 (31%)	
	6	5 (11%)	2 (7%)	3 (20%)		3 (8%)	2 (29%)		2 (12%)	3 (10%)	
	7	1 (2%)	1 (3%)	0 (0%)		1 (3%)	0 (0%)		0 (0%)	1 (3%)	

ELS = elective laparoscopic surgery; ULS = urgent laparoscopic surgery; PRE/EA-Puberty = prepubertal/early puberty; MI/LA-Puberty = middle/late puberty.

## Data Availability

Data supporting reported results are archived in the first author’s personal datasets.

## Supplementary Materials

The following supporting information can be downloaded at: https://www.mdpi.com/article/10.3390/children10111771/s1, Table S1: Scoring system details.
