# Supplementary material for: Laparoscopic Cholecystectomy in Children: The Experience of Two Centers Focusing on Indications and Timing in the Era of “New Technologies”
Source: Children (Basel). 2023 Oct 31;10(11):1771. doi: 10.3390/children10111771 (PMC10670818; doi:10.3390/children10111771)
Supplement: Supplementary file 1 [file children-10-01771-s001.zip › children-2647812-supplementary.pdf]

Table S1: The detailed scoring system

Variables	***Specific features***
Clinical	- symptom duration > 7 days<br>- local Murphy sign<br>- concomitant hematological disease<br>- overweight / obesity<br>- previous lithotherapy
Ultrasounds	- multiple calculi<br>- sonographic Murphy sign<br>- sliding calculus<br>- pericholecystic fluid<br>- biliary tree calculi<br>- gallbladder wall thickening > 3 mm<br>- gallstone > 3 mm<br>- gallbladder distension<br>- biliary tree calculi/biliary tree dilatation<br>- hepato/splenomegaly<br>- lymph nodes in the hepatic pedicle
Surgical	- gallbladder adhesions >50%<br>- gallbladder distension<br>- gallbladder unable to grasp<br>- stone impact<br>- signs of inflammation<br>- time to identify cystic artery/duct > 90min
Histological	- Ulcers and/or erosion<br>- Inflammatory cell infiltration<br>- Fibrosis<br>- Adenomyosis<br>- Reactive epithelial hyperplasia<br>- Epithelial atrophy<br>- Parietal atrophy<br>- Intramural microlitiasis<br>- Intestinal metaplasia

For each parameter, a dichotomous variable was created (0 = absence; 1 = presence); for histological features, a severity grade (1-2-3) was further added
